# Effects of diabetes-induced hyperglycemia on epigenetic modifications and DNA packaging and methylation during spermatogenesis; A narrative review

**DOI:** 10.22038/IJBMS.2023.69604.15173

**Published:** 2024

**Authors:** Aram Minas, Mariana Camargo, Marco G. Alves, Ricardo Pimenta Bertolla

**Affiliations:** 1 Department of Surgery, Division of Urology, Human Reproduction Section, São Paulo Federal University, São Paulo, Brazil; 2 Department of Anatomy and UMIB - Unit for Multidisciplinary Research in Biomedicine, ICBAS - School of Medicine and Biomedical Sciences, University of Porto, Porto, Portugal

**Keywords:** Diabetes mellitus type 1, DNA, DNA damage, Epigenomics, Spermatogenesis

## Abstract

The impact of diabetes on various organs failure including testis has been highlighted during the last decades. If on one hand diabetes-induced hyperglycemia has a key role in induced damages; on the other hand, glucose deprivation plays a key role in inducing male infertility. Indeed, glucose metabolism during spermatogenesis has been highlighted due to post-meiotic germ cells drastic dependence on glucose-derived metabolites, especially lactate. In fact, hyperglycemia-induced spermatogenesis arrest has been demonstrated in various studies. Moreover, various sperm maturation processes related to sperm function such as motility are directly depending on glucose metabolism in Sertoli cells. It has been demonstrated that diabetes-induced hyperglycemia adversely impacts sperm morphology, motility and DNA integrity, leading to infertility. However, fertility quality is another important factor to be considered. Diabetes-induced hyperglycemia is not only impacting sperm functions, but also affecting sperm epigenome. DNA packing process and epigenetics modifications occur during spermatogenesis process, determining next generation genetic quality transmitted through sperm. Critical damages may occur due to under- or downregulation of key proteins during spermatogenesis. Consequently, unpacked DNA is more exposed to oxidative stress, leading to intensive DNA damages. Moreover, epigenetic dysregulation occurred during spermatogenesis may impact embryo quality and be transmitted to next generations, increasing offspring genetic issues. Herein we discuss the mechanisms by which diabetes-induced hyperglycemia can affect epigenetic modifications and DNA packaging and methylation during spermatogenesis thus promoting long-lasting effects to the next generation.

## Introduction

The world health organization (WHO) defines infertility as a health condition in which couples cannot achieve a clinical pregnancy after 12 months of unprotected intercourse (1). Infertility has become a worldwide health issue affecting 15% (48.5 million couples) of reproductive-age couples, in which male infertility is present in almost 50% of infertile cases, with 20% of unknown reasons (2). Thus, it is essential to unveil the causes of infertility in these couples. 

Diabetes is well-known as a metabolic disorder characterized by associated hyperglycemia with disrupted insulin production or activity (3). Considering the international diabetes federation (IDF) characterization, diabetes is divided into three different subtypes: type 1 (T1D), 2 (T2D), and Gestational. In 2019, diabetes prevalence, regardless of type, was estimated at 463 million people worldwide. In addition, an IDF epidemiological study predicted that this number will continue to increase in the following years, rising to 578 million by 2030, affecting 10.2% of the worldwide population (4).

Increased blood glucose level is defined as hyperglycemia, which is a key factor in diabetes diagnosis. Persistent hyperglycemia is the main characterizing factor for prediabetes and type 2 diabetes diagnosis (5, 6) and results from cellular insulin resistance or insufficient insulin secretion from pancreatic B-islets to face blood glucose levels (5, 6). Hyperglycemia can be diagnosed by analyzing fasting blood glucose levels or glucose tolerance. The impaired fasting blood glucose levels (IGF) are defined by blood glucose concentration higher than 7mMol/L. In contrast, impaired glucose tolerance (IGT) is a condition where glucose tolerance is higher than 11 mmol/ l2 hr after a 75 g oral glucose consumption. These two parameters have been used for diabetes mellitus (DM) diagnosis (7). In corroboration, it has been reported by several clinical studies that T1D-induced hyperglycemia adversely impacts semen quality. Indeed, it can cause a significant reduction in semen volume (8) and sperm morphology (9), concentration (8), and motility (10). In addition to that, T2D is also known to disrupt normal erectile and ejaculation functions (11). Therefore, hyperglycemia is known as one of the main factors causing infertility, with chronic and silent features impacting the male reproductive system (12). Indeed, various previously conducted studies illustrated the potential of hyperglycemia in inducing oxidative stress (13, 14), inflammation (14), intrinsic apoptosis (13), and epigenetic dysregulation (15) in testicular tissue resulting in decreased sperm quality and DNA integrity (16). This is of utmost relevance due to the essential role of DNA integrity and epigenetic modifications in male fertility with a direct impact on overall progeny health. This narrative review will discuss the state-of-art studies regarding diabetes-induced hyperglycemia, as a growing infertility-inducing factor, and its effects on sperm DNA packaging and epigenetics modifications during spermatogenesis.

***Review criteria***

This study was conducted with studies published in English. The selected criteria were: Original study, Clinical Study, Comparative Study, Journal Article, Evaluation Studies, Multicenter Study, and Meta-Analysis. Congresses Dataset, Congresses Presentations, Letters, Introductory Journal Articles, Observational Studies, and studies not published in English were excluded. 

The following keywords were searched: ‘Hyperglycemia’ OR ‘Blood Glucose’ OR ‘Insulin’ OR ‘Male Infertility’ OR ‘Testis’ OR ‘Spermatogenesis’ OR ‘Sperm’ OR ‘Semen’ OR ‘DNA’ OR “diabetes” OR “spermatogenesis” OR “methylation” OR “DNA fragmentation” OR “histones” in combination with other search phrases relevant to the topic of hyperglycemia used. 

***Effect of hyperglycemia on sperm parameters***

A condition described as “collapse of the sexual functions” in men was reported in the 11th century referring to a situation in which the male partner suffered from sexual dysfunction and infertility due to their dysregulated glucose metabolism (9). Later, in 1975, Zysk and colleagues explained the issue more precisely by showing spermatogenesis directly depends on glucose uptake and consumption, even in low concentration, in order to maintain physiological sperm production (17). Although it was suggested that hyperglycemia is age-dependent, it has currently been consistently discussed how this condition also occurs in children, adolescents, and men of reproductive age (3, 18). La Vignera and colleagues showed that almost 50% of patients with diabetes exhibit sub/infertility that was correlated with hyperglycemia (19). Various studies illustrated the T1/2D-induced hyperglycemia’s adverse effects on male reproductive function and birth rate (20, 21). For instance, retrograde ejaculation (22), erectile dysfunction (11), endocrine disruption, and altered sperm quality have been reported in diabetes-induced hyperglycemia (9). 

A study demonstrated that loss of glucose control results in decreased sperm motility only without alterations in any other parameter of sperm quality (23). Another study showed that sperm chromatin damage is increased in T1D-induced hyperglycemia (8). Finally, a semen proteomic study demonstrated altered proteomic profile in hyperglycemic T1D men compared to normoglycemic individuals. Results indicated the accumulation of modified forms of the eppin (epididymal proteinase inhibitor) protein complex (EPC) components semenogelin-1, clusterin, and lactotransferrin in the sperm proteomes, all of which present important roles in fertilization (24). Moreover, different studies illustrated negative impacts on hormonal balance during T1D-induced hyperglycemia (25, 26). Indeed, research showed that this dysregulation might be due to the impact of hyperglycemia on the hypothalamus-pituitary-gonadal (HPG) axis (25). However, testosterone, LH, and FSH did not change in the absence of concomitant diseases. On the other hand, T1D-induced hyperglycemia in association with neuropathy or renal glomerulopathy led to reduced blood androgen levels (25, 26).

Considering that obesity might lead to a prediabetes state - including hyperglycemia - Crisóstomo and colleagues illustrated that high-fat diet (HFD)-induced prediabetes results in long-term and irreversible impacts on spermatogenesis. The results emphasized that metabolic changes may be reversed with diet, but irreversible HFD-induced prediabetes effects can occur in sperm quality (i.e., sperm count, motility, and viability) (27). Later, it was shown that mice under the HFD regime (for 200 days) from day 21 after birth until adulthood developed prediabetes and showed irreversible lipid damage and testicular lipid content and metabolism changes, resulting in a permanent decrease in sperm quality (28). On the other hand, Crisóstomo and colleagues later reported that those metabolic changes and the decreased sperm quality not only affect the parents but are also inheritable and can adversely affect F1 and F2 male generation testicular metabolome and even their sperm quality (28-30). These studies highlight that lasting hyperglycemia, even if moderated, causes permanent effects on male reproductive health that can even pass to the next generations.

***Glucose metabolism in testicular tissue***

Testicular somatic and germ cells have several metabolic features based on their specific metabolic requirements, which impacts the blood and somatic cells’ metabolic supply network with germ cells (31). Testicular tissue, like any other tissue in the body, requires glucose as the primary energy source for homeostasis. However, testicular germ cells’ metabolism changes during the spermatogenesis process from aerobic (in spermatogonia) to an anaerobic (post-meiotic germ cells) pathway (32). In line, it has been revealed that basal compartment cells, such as spermatogonia, use glucose as an energy source to produce ATP (33). On the other hand, lactate consumption starts in spermatocytes and during later stages becomes the primary energy source for germ cells. Although lactate plays the main role in post-meiotic germ cells, it should be noted that all glycolytic enzymes are present in germ cells, and spermatozoa metabolism depends on glycolytic pathway activity and it uses glucose and fructose as the main energy source (32). Moreover, it has been revealed that lactate is not only involved in spermatogenesis as an energy source but also plays a key role in spermatid RNA and protein synthesis (34). In addition, lactate exposure exhibited protective effects on spermatogenesis by inhibiting germ cell apoptosis (35) and stimulating the spermatogenesis process (36).

Considering the intensive need and use of lactate in germ cells, Sertoli cells convert glucose to lactate and thus provide testicular germ cells with this key energy source (37). Sertoli cells import glucose using glucose transporters known as Glut proteins. Indeed, the Sertoli cells express five types of Glut 1, 2, 3, 8, and 4 proteins in order to uptake glucose from the interstitial tissue, converting them to lactate using lactate dehydrogenase (38). The regulatory mechanism responsible for glucose conversion to lactate is multi-complex, which requires various essential factors to maintain this mechanism through different pathways. Testosterone and FSH, for example, are recognized as hormonal factors involved in glucose uptake and lactate production (39), while insulin-like growth factors (IGF I and II), insulin receptors (INSR), and type-I insulin-like growth factor receptor (INS1R) are other factors involved, due to their function in inducing Glut protein expression in Sertoli cells (40). Moreover, it has been shown that IGF1 and INS1R participate in Sertoli cells’ energy metabolism through their involvement in nucleotide (ATP, GTP, UTP) uptake. In addition, these factors are involved in transferrin, pyruvate, and lactate secretion, supporting the key role of insulin in regulating physiological spermatogenesis (41). 

Sertoli cells then transport lactate to the intra-tubular fluid, where it can be used by developing germ cells through specific lactate transporters known as monocarboxylate transporters (MCT). Different studies observed that MCT 1 and 4 play a key role in transferring these proteins to the intra-tubular fluid where germ cells are located (42, 43), supporting the main idea of germ cells’ lactate consumption priority in post-meiotic stages (31). Indeed, lactate production is important, so Sertoli cells appear to adapt their metabolism through stress conditions such as glucose (44) or insulin deprivation (45). 

Sertoli cells convert their metabolism from catabolic to anabolic through the AMP-activated protein kinase (AMPK) signaling pathway in glucose deprivation conditions (46, 47). AMPK pathway activation directly impacts Sertoli cells’ lactate production and secretion by increasing Glut1 and MCT4 proteins expression (48). Moreover, using knock out (KO) mice models, it has been revealed that AMPKα1^-/-^ results in reduced sperm motility, mitochondria dysfunction, and morphological impacts on the sperm head (49). It has been revealed that AMPK up-regulates nicotinamide adenine dinucleotide-dependent type III deacetylase sirtuin 1 (Sirt1) activity (50). Sirt1 plays a key role in germ cell survival, glucose metabolism in testicular tissue, and mitochondria respiration. For instance, Sirt1 gene deletion results in disruption of spermatogenesis (51). AMPK in association with Sirt1 maintains and regulates mitochondrial biogenesis (50) and in association with PGC-1 regulates oxidation of fatty acids, ATP synthesis, and lipid homeostasis (52). Indeed, it has been revealed that energy sensors such as AMPK are primary sensors activated during stress (53, 54). 

Hyperglycemia in T1D and its impact on Sertoli cell function is also a critical issue. Consistent with this, it has been revealed that T1D-induced hyperglycemia directly affects Sertoli cell production of growth factors, such as glial cell line-derived neurotrophic factor (GDNF). Moreover, T1D-induced hyperglycemia reduced GDNF receptor expression in rat testicular tissue (Gfrα1 and c-Ret). Consequently, reduced GDNF and GDNF-receptors resulted in diminished spermatogonial stem cells (SSCs) self-renewal (55). Moreover, it has been revealed that testicular Sertoli cell fibroblast growth factor 21 (FGF21) is involved in germ cell survival during T1D-induced hyperglycemia. Decreased FGF21 led to testicular germ cell apoptosis. However, FGF21 treatment could ameliorate this condition by increasing AMPK/Sirt1/ PGC-1 pathway activity, resulting in normal energy metabolism in germ cells (56). 

***Hyperglycemia-related male infertility and spermatogenesis failure in animal models***

It has been revealed that T1D causes a significant decrease in seminiferous tubule diameters and a remarkable increase in epididymis lumen size (57). T1D-induced hyperglycemia causes numerous pathological impacts on the testis, including germ cell dissociation and depletion, reduced positive tubular differentiation index (TDI) percentage, increased seminiferous tubules number with negative spermiogenesis index (SPI), diminished repopulation index (RI) percentage, and down-regulated Leydig, spermatogonia A and B, and Sertoli cells number *per* mm^2^ in testis (55, 58). Moreover, authors have demonstrated, in corroboration with previous studies, a significant decrease in body and testicular weight, lower testicular and epididymal sperm content, and decreased sperm motility (57, 59). On the other hand, Amaral *et al*. in 2006 reported lower sperm concentration and motility in Goto-Kakizaki (GK) diabetic rats (60). These data corroborated findings by Azarniad *et al*. in 2020 (55), which reported reduced sperm parameters in T1D-induced rats using Streptozocin (STZ). However, STZ-induced T1D rats showed sex behavior changes and lower gonadal weight (61). Accordingly, it has been revealed that uncontrolled blood glucose (hyper/hypo-glycemia) leads to catastrophic damage to testicular physiological function (62). In the case of hyperglycemic rats, steroidogenesis disruption (63) resulted in a 25-fold decrease in serum testosterone levels and reduced birth rate after mating (64). Indeed, reduced testosterone synthesis has been shown to be associated with increased testicular glycogen synthesis, exhibiting a direct/indirect influence of hyperglycemia on testicular metabolic and hormonal disruption in T2D-induced hyperglycemia (65). Moreover, the reduced activity of phosphofructokinase 1 and lactate dehydrogenase (LDH) leads to increase cellular glucose uptake and reduces lactate production (65). In corroboration with this molecular evidence, it has been reported that Sertoli cell number and activity are reduced in STZ-induced hyperglycemia in induced T1D, suggesting increased glucose uptake is not only associated with decreased phosphofructokinase 1 and LDH but also might be associated with reduced Sertoli cell number in testicular tissue (55). Accordingly, hyperglycemia caused testicular damage by altering different cellular and molecular mechanisms in somatic or germ cells. Thus, Sertoli cell alterations might generate numerous impacts on testicular seminiferous tubules’ structural integrity and germ cells’ cellular and molecular maintenance. 

***Hyperglycemia-induced oxidative stress and spermatogenesis failure in animal models***

It has been discussed that hyperglycemia, whether induced by STZ or a high-fat diet, in animals, results in dramatic testicular anti-oxidant defense and increased reactive oxygen species (ROS) generation (12, 66). Indeed, Samadian and colleagues in their recent study illustrated that testicular total anti-oxidant capacity (TAC), superoxide dismutase (SOD), and glutathione peroxidase (GPX) enzyme levels dramatically decrease in T1D-induced hyperglycemia in rats. However, insulin therapy could preserve testicular redox balance against T1D-induced hyperglycemia, indicating that hyperglycemia directly impacts the redox homeostasis in testicular tissue (66). Moreover, T2D-induced hyperglycemia also directly impacts mitochondria anti-oxidant defense (67) and biogenesis (68) in testicular cells. Indeed, even mild hyperglycemia led to decreased mitochondrial DNA content, resulting in down-regulation of respiratory capacity and ROS overproduction (69). In T1D-induced hyperglycemic rats, pro-apoptotic factors such as Bax, Bad, and c-Jun N-terminal kinase upregulation were also reported, which was correlated with an increase in germ cell death (70, 71). Moreover, more recently conducted studies reported a dramatic down-regulation of Bcl-2 protein in T1D-induced hyperglycemia (58, 72). However, these studies showed that insulin therapy could positively diminish blood glucose levels in rats leading to reduced hyperglycemia impacts on testicular tissue. Results illustrated a positive impact of insulin therapy on modulating mitochondria-dependent apoptosis (58, 66). 

Accordingly, in a recently conducted study, T1D-induced hyperglycemia in rats led to oxidative stress-induced DNA damage in testicular tissue, resulting in p53 and p21 activation during spermatogenesis. Consequently, this alteration led to p53-dependent apoptosis and p21-induced cell cycle arrest in G1 to S phase during germ cell mitotic division. At the same time, insulin co-treatment preserved testicular tissue and spermatogenesis from the deleterious hyperglycemia-induced effects of oxidative stress on germ cells leading to higher sperm production and sperm motility (58). 

It has also been demonstrated that hyperglycemia-induced oxidative stress, even in mild hyperglycemia, can negatively impact epididymal sperm content in pre-diabetic rats (73) leading to sperm membrane lipid peroxidation (74) and oxidative stress-induced DNA fragmentation in sperm (58). Hyperglycemia-generated ROS is known as one of the main mechanisms by which DM impacts male reproductive health, and ROS overproduction was detected in the semen of men with diabetes (75). In the case of T1D-induced hyperglycemia, it has been shown by various studies that semen quality, total oxidant levels, and testicular anti-oxidant levels are all altered (66), illustrating that oxidative unbalance is an important consequence of testicular hyperglycemia. Indeed, this has been supported by multiple successful original studies using anti-oxidative agents to rescue hyperglycemia-induced oxidative damage in the male reproductive tract (64, 76). Thus, according to these studies, it is possible to observe that hyperglycemia control in T1/2D has a positive impact on spermatogenesis failure. 

***Hyperglycemia impact on histone modification, DNA packing, and epigenetic modifications***

It is well known that, in normal spermatogenesis conditions, germ cell histones are replaced by transition proteins (TPs), which are then replaced by protamines (77). Indeed, chromatin condensation plays a crucial role in maintaining DNA integrity and protecting from free radicals and carbonyl group-associated DNA fragmentation (78). This process will not be complete unless chaperones actively process the transition. In testicular somatic and germ cells, testicular specified chaperones regulate nucleosomal assembly. They are described as the key regulators of histone and non-histone genome organization (OR genome-condensing assembly) (79). Indeed, chaperone heat shock proteins (HSPs) also play a critical role during DNA packaging and methylation. These proteins have different structures and weights, which have been described in 20 family members (79). HSPs participate in quality control during cell differentiation and growth in many tissues (80). In the testes, only Hsp70 and Hsp90 have been identified (81). These proteins not only play a key role in inducing/inhibiting the apoptosis process in testicular germ cells (82, 83) but also significantly interact with TPs and post-meiotic chromatin condensation (84, 85), demonstrating an important role in sperm maturation which will be further explained. 

***Effects on DNA packaging***

In the case of diabetes-induced hyperglycemia, Bahmanzadeh and colleagues in 2019 showed a significant decrease in sperm chromatin condensation in T1D-induced hyperglycemia. Their results indicated that sperm chromatin condensation significantly decreased, and sperm DNA damage increased in T1D-induced rats compared to controls. On the other hand, in the same study, when T1D-induced rats were treated with 5 mg/kg of resveratrol (an anti-oxidant agent) chromatin condensation and DNA integrity were preserved. Thus, the authors concluded that DNA packaging and integrity might be affected by hyperglycemia-induced oxidative stress in rats with T1D (86). Researchers in 2017 showed that the protamine1/protamine 2 ratio was altered in T2D-induced mice with hyperglycemia when compared to control animals, further illustrating chromatin condensation alterations in hyperglycemic mice. Protamine 1 mRNA expression increased significantly in these animals when compared to normoglycemic controls. Moreover, sperm head morphology, pregnancy rate, and number of both male and female offspring reduced in F1 and F2 generations post T2D-induced hyperglycemia in fathers, suggesting that T2D-induced hyperglycemia causes intergenerational and transgenerational effects on the reproductive health of the offspring (87). Accordingly, in a recently conducted study, Aeeni *et al*. reported a dramatic decline in the level of Hsp70, TP1, and TP2 gene expression and protein level in testicular tissue of T1D-induced hyperglycemic rats. Moreover, their results are corroborated with those previously mentioned, demonstrating a significant decline in mature sperm number in hyperglycemic rats when compared to controls. On the other hand, insulin treatment decreased this negative impact of hyperglycemia both in testicular tissue and sperm (12). 

Increased DNA fragmentation and alteration of post-meiotic maturation in sperm have long been recognized and well-discussed to affect fertilization potential (88, 89). Accordingly, it seems that hyperglycemia results in the down-regulation of Hsp70, TP1, and TP2 proteins could negatively impact DNA packaging during spermatogenesis. Although there is no specific study reporting the impact of diabetes-induced hyperglycemia on histone modifications, it has been revealed that histone acetylation occurs in round spermatids and before histone replacement during spermatid elongation at stages V-VIII (79). Considering that Hsp70 expression is increased in the round and elongated spermatids, and directly associates with TP1/2 by forming an acid-resistant complex (12, 79), it is possible to conclude that diabetes-induced hyperglycemia indirectly reduces chromatin condensation by reducing Hsp70 expression. Accordingly, there is a positive correlation between Hsp70 and both TP1 and TP2 mRNA levels (85). Thus, it can be suggested that diabetes-induced hyperglycemia not only impacts DNA packaging and methylation by dysregulating the histone modification process but also directly disrupts DNA maturation and TPs/protamines replacement by inhibiting Hsp70 and TP1/2 expression (Figure 2). Moreover, reduced protamine 1/protamine 2 ratios could amplify this defect and have an indirect effect on spermatozoa. Indeed, altered DNA packaging can potentiate DNA damage in spermatozoa due to ROS. Increased DNA damage can consequently result in infertility. It has been revealed that insulin therapy can significantly ameliorate hyperglycemia-induced DNA packaging defects. However, it is still unknown if this defect will not be transmitted to the next generation, considering it has been reported that at least some of these defects can be inherited (79). 

***Effects on DNA methylation***

DNA methylation in testicular tissue occurs simultaneously during spermatogenesis in order to transmit the epigenetic information from the father to the next generation (90). Alteration in epigenetic organization (such as DNA methylation disruption) may lead to important consequences for male fertility, such as impaired embryo development (91, 92). This process consequently impacts the transcription and gene expression (91) and/or alters the DNA integrity at later embryonic stages (93). Bose and colleagues (2012) revealed that DNA methylation was not impacted in preleptotene/zygotene cells, even after 2 spermatogenetic cycles in diabetes-induced hyperglycemia mice. However, labeling methylation index (5 methyl cytosine staining index) in testis showed no statistically significant difference for hypermethylation in testes after 36 and 72 days of hyperglycemia when compared to control animals (94). Although Bose and colleagues suggested that global methylation was not impacted during spermatogenesis, El-Behery and coworkers recently revealed that DNMT3a (DNA methyltransferase 3a) – a methylation involved enzyme and *de novo* methylation controller during germ cells division – was altered in induced hyperglycemia. Indeed, these results suggested that diabetes-induced hyperglycemia led to down-regulation in DNMT3a activity in spermatogonia and spermatocytes of T1D-induced diabetic rats when compared to controls (95). 

Accordingly, it has been revealed that DNMTs directly enhance Histone deacetylases (HDACs) and down-regulate Histone acetyltransferases (HATs) expressions. Considering DNMT3a is down-regulated in diabetes-induced hyperglycemia, it might be expected that this reduction in DNMT3a expression results in HDAC enzyme down-regulation and HAT enzyme up-regulation, leading to hypomethylation. Although this has not yet been demonstrated, Aeeni et al. reported a significant testicular tissue global DNA hypomethylation during spermatogenesis in diabetes-induced hyperglycemic rats (12). The Aeeni *et al*. study is the first to discuss the direct impact of hyperglycemia on DNA methylation status during spermatogenesis. However, the main questions that still stand are: (i) whether this hypomethylation status is an impact of hyperglycemia-induced oxidative stress or testosterone withdrawal; and (ii) if there is a direct correlation between metabolic pathway and DNA methylation during spermatogenesis. In more detail, the same hypomethylation condition has been illustrated in clinical and research studies conducted on varicocele (85, 93). However clinical studies suggested this might be an impact of ROS in varicocele (VCL) (93, 96). A study revealed that exogenous testosterone therapy could positively improve global DNA methylation in varicocele-induced rats (85). Thus, in diabetes-induced hyperglycemia conditions, this should be verified whether this improvement is related to testosterone restoration or ROS down-regulation. 

Moreover, El-behery and colleagues in 2019 showed a PCNA^+^ (Proliferating cell nuclear antigen) cell number reduction in diabetes-induced hyperglycemia condition, which led to the conclusion that there is a reduced DNA repair mechanism in diabetic rat testicular tissue (95). However, recent reports revealed a distinct role of this protein in maintaining and controlling DNA replication, maintenance, and specific DNA methylation during spermatogenesis (85, 97). Moreover, it has been revealed that PCNA interacts with DNMT1 (98) and directly increases DNMT1 expression two-fold (98, 99). Considering these results, it might be relevant to suggest that PCNA-related DNA methylation might be one of the possible pathways in the dysregulation of DNA methylation in induced hyperglycemia during spermatogenesis. In corroboration with previous reports that indicated that the impact of diabetes on epigenetic dysregulation specifically during spermatogenesis led to infertility (100) that can be inherited in oﬀspring (15), according to these findings, we can suggest that diabetes alters methylation. However, in order to demonstrate this impact, further studies are necessary. 

**Figure 1.**
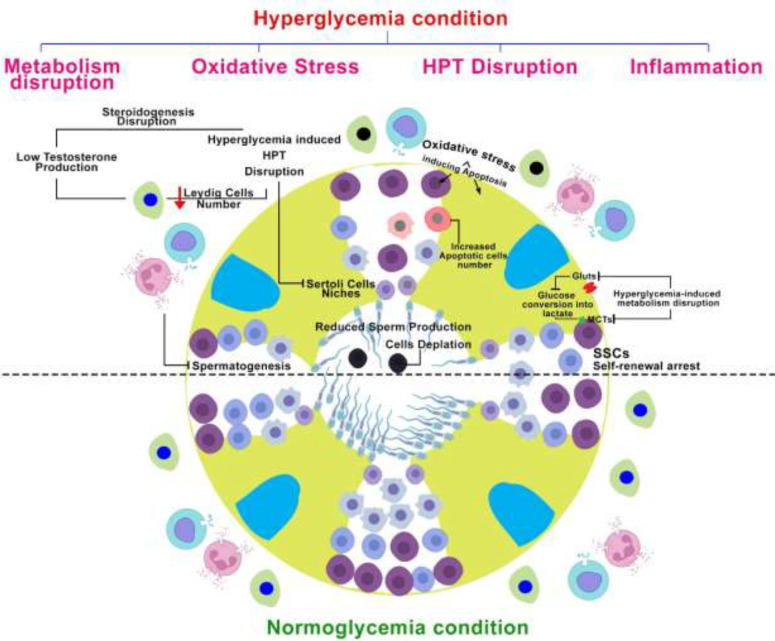
Schematic figure presents diabetes-induced hyperglycemia impacts on male reproductive germ cells

As shown, hyperglycemia disrupts the hypothalamus-pituitary-testis axis as well as testicular physiological glucose metabolism. Testicular steroidogenesis disruption, oxidative stress, and inflammation, as pathophysiological effects of hyperglycemia result in Sertoli cell dysfunction, germ cell loss, and reduced sperm production and motility.

**Figure 2:**
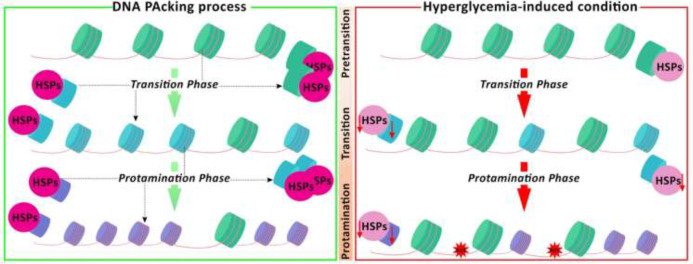
Presented graphical abstract illustrates the diabetes-induced hyperglycemia impacts on the DNA packing process during spermatogenesis

Hyperglycemia impacts all three stages of DNA packing including pre-transition, transition, and protamination processes by reducing not only testicular heat shock proteins (HSPs) but also by diminishing transition proteins and protamine expression during spermatogenesis. Consequently, the sperm DNA condensation and packing process will not be fully accomplished in the hyperglycemia condition, making DNA more susceptible to facing oxidative stress.

## Conclusion

Diabetes is dramatically increased among children, adolescents, and young adults. The current literature highlights the negative impact of this metabolic disease on male fertility. Indeed, diabetes-induced hyperglycemia is a critical condition that impacts testicular spermatogenesis and sperm production and maturation. Insulin treatment appears to have a positive effect on male fertility, although further studies are needed to evaluate how long-lasting treatments can impact spermatogenesis and/or fertility potential. Notably, hyperglycemia may directly impact DNA packaging and epigenetic modifications during spermatogenesis. It has been revealed that this dysfunction might be linked to decreased levels of various proteins, such as TPs, protamines, and DNMT3a. However, there is still a low number of studies that demonstrated these underlying mechanisms. Thus, considering the direct association between DNA packaging and methylation with sperm DNA integrity, as well as DNA epigenetics with embryo development, it is important to further expand studies on these subjects to unveil the molecular mechanisms by which hyperglycemia affects all these processes. Based on the increased prevalence of diabetes worldwide, the demonstrated mechanisms are an important step toward proposing a solution to the inheritable epigenetic-induced dysfunctions in diabetes-induced hyperglycemic men. Recent studies highlight the potential of hyperglycemia to affect the offspring, thus propagating metabolic dysfunctions and/or infertility. Therefore, it is essential to further study the possible inter and transgenerational effects of hyperglycemia and diabetes. In addition, different studies seem to demonstrate that insulin therapy might help to restore the testicular environment. However, more studies should be performed in order to verify the dose and how the medication shifts back to homeostasis. 

## Conflicts of Interest

The authors declare no conflicts of interest.
